# miR-26b inhibits isoproterenol-induced cardiac fibrosis via the Keap1/Nrf2 signaling pathway

**DOI:** 10.3892/etm.2020.9412

**Published:** 2020-10-29

**Authors:** Shaohua Xiang, Jing Li, Zhengfu Zhang

Exp Ther Med 19:2067-2074, 2020; DOI: 10.3892/etm.2020.8455

After the publication of the above paper, the authors have realized that the first author’s name was spelt incorrectly as “Shaohua Xian”. The correct spelling (“Shaohua Xiang”) is presented above. Furthermore, the affiliation for Shaohua Xiang was also incorrect (the Hospital’s name was featured incorrectly); this should have been given as “Department of Cardiothoracic Surgery, **Dianjiang County Hospital of Traditional Chinese Medicine**, Chongqing 408300, P.R. China”. Therefore, the authors’ names and affiliations in this paper should have been presented as follows:

SHAOHUA XIANG^1^, JING LI^2^ and ZHENGFU ZHANG^2^

^1^Department of Cardiothoracic Surgery, Dianjiang County Hospital of Traditional Chinese Medicine, Chongqing 408300; ^2^Department of Cardiothoracic Surgery, People’s Hospital of Changshou, Chongqing 401220, P.R. China

The authors regret that this error with the author affiliations was not noticed prior to the publication of their paper, and apologize for any inconvenience caused.

